# A Case of Fatty Degeneration of the Placenta, Causing Abortion in a Lady Habitually Subject to Premature Delivery; with Remarks

**Published:** 1841-01-30

**Authors:** A. J. Wedderburn

**Affiliations:** U. S. Navy


### ORIGINAL COMMUNICATIONS.

*A Case of Fatty Degeneration of the Placenta, causing abortion in a Lady habitually subject to premature delivery; with remarks.* By A. J. WEDDERBURN, M. D., of the U. S. Navy. *Read before the Pathological Society of Philadelphia, by* THOMAS D. MUTTER, M. D.

A LADY, residing near Pensacola, has been so unfortunate as to have had eight or nine premature births; the period has several times been extended to the seventh and eighth month, but in every case except the first the child has been born dead. She attributes the first case to an injury received from a fall.

For one who has borne so many children, she has a remarkably healthy appearance, and enjoys very good health, except occasionally being troubled with dyspeptic symptoms.

On being called to see her during her last pregnancy, she informed me that she had reason to believe the fœtus was dead, relating the usual symptoms in such cases. I learned that she had been attended by different medical men in almost every case; that no particular attention had been paid by any of them to the causes producing the death of the fœtus and premature births; that the children were all dropsical, and that all who had attended her, and all she had consulted both in this country and in France, had advised her to remain quiet, and take no exercise during the full period of her pregnancy.

About ten days after my first visit, I was called again, and unfortunately found that her fears were well founded. The labour was a simple one, and was completed in a short time. The period was between seven and eight months. On delivering the placenta, I was immediately struck with its immense weight and size. I did not weigh it, but its weight was nearly or fully equal to that of the child, the child, at the same time, being very well grown. You may be surprised when I tell you (for I have not been able to find on record a case of obesity of the placenta) that all that part of it that is attached to the uterus was filled with fatty matter, having the appearance of the fat found in the duplicatures of the peritoneum. This mass of fat was more than an inch in thickness.

I examined the child, and found it perfectly formed, but there was a serous effusion in the sub-cutaneous cellular membrane, and in all the cavities of the body.

It has been generally the case with this lady to suffer very much with œdema of the lower extremities, after the time of quickening. With regard to the condition of the placenta, in former cases, I could gain no information, no attention having been paid to its condition before.

I am convinced, from reflecting on the subject, that the advice she has received has been contrary to what it should have been, and probably what would have been directed in the case, had those whom she has consulted been acquainted with the facts that have been lately developed. I think there can be no doubt that the absolute rest that has been enjoined on her has induced a degree of plethora, and a deposit of fat in the placenta, which has retarded the fœtal circulation and produced the disease with which they have all died.

Now the plan of treatment that suggests itself to me, in a case of this kind, is regular and moderate exercise during the full period of pregnancy, strict attention to a light diet, and in order to more effectually guard against a recurrence of the diseased state of the placenta, it would be advisable to resort to the treatment prescribed by Dewees in cases of deformed pelvis, after the period of quickening, i. e. the use of the lancet once a month, and the administration of squills, digitalis, and calomel.

This lady is now pregnant, period four months; was threatened about a week since with an abortion; bled her and gave acetate of lead and opium; she is now entirely relieved, but takes tinct. digitalis. I will also mention here, that she has, during the last four or five months, suffered at times with hemicrania, for which I am about to commence the use of the carb. ferri.

This case is very interesting from its rarity, but it is not, we believe, entirely unique: for, although the vascular connexion between the uterus and fœtus, through the medium of the placenta, and the exact functions of the latter may be still unsettled, yet its liability to many of the morbid alterations found in other tissues and a consequent predisposition to abortion, are placed beyond a doubt. Thus cases of hypertrophy and atrophy, softening carried to pulpiness; and induration to the extremes of a cartilaginous and even ossified state, are not wanting, while hydatids, and a scirrhous degeneration are still more frequent. The alteration detected by Dr. Wedderburn is also noticed, but not at length. Jourdan states, that among other changes, there is occasionally to be found, in the parenchyma of the placenta, *a true steatomatous deposit*, but he enters into no details: the observation, therefore, of Dr. W. (although the weight of the placenta, the proportional amount of fatty matter, and its analysis are unfortunately wanting) is the most explicit we have seen. We trust its publication will serve to elicit further information upon a point which is certainly not devoid of interest.—Eds. Med. Exam.

